# Impact of incorporating spent oil filtering earths into the formulation of alkali-activated cements based on electric arc furnace slag

**DOI:** 10.1007/s11356-025-36429-w

**Published:** 2025-04-24

**Authors:** Pedro Delgado-Plana, Miguel Ángel Gómez-Casero, Salvador Bueno-Rodríguez, Pedro José Sánchez-Soto, Dolores Eliche-Quesada

**Affiliations:** 1 Department of Chemical, Environmental, and Materials Engineering, Higher Polytechnic School of Jaén, University of Jaen, Campus Las Lagunillas S/N, Jaén, 23071 Spain; 2 Center for Advanced Studies in Earth Sciences, Energy and Environment (CEACTEMA), University of Jaén, Campus Las Lagunillas, S/N, Jaén, 23071 Spain; 3 Institute of Materials Science of Sevilla, Joint Center of CSIC (Spanish National Research Council) and University of Sevilla, Seville, 41092 Spain

**Keywords:** Spent oil filtering earths, Electric arc furnace slag, Alkaline-activated cements, Mechanical properties, Thermal conductivity, Microstructure

## Abstract

In this study an investigation of the effect of incorporating spent oil filtering earths (SOFE) as a precursor in the manufacture of alkaline activation cements based on electric arc furnace slags (EAFS) has been carried out. SOFE were mixed up to 50 wt% with EAFS at 10 wt% intervals. As a control, a binder containing only EAFS was manufactured. The fresh binder samples were cured at room temperature for 7 and 28 days. Phase analysis was conducted using X-ray Diffraction (XRD), attenuated total reflectance Fourier transform infrared spectroscopy (ATR-FTIR), and Scanning Electron Microscopy-Energy Dispersive X-ray spectroscopy (SEM–EDS). The results indicated that the addition of SOFE caused a delay in the geopolymerization or alkaline activation reactions, which resulted in a decrease in mechanical properties at low hydration times, 7 days. However, substitution of SOFE led to an improvement in physical, mechanical, and thermal properties after 28 days of curing. Ideal substitution ratios were 30 wt% or higher. At optimum substitution ratios, the bulk density of alkaline-activated cements decreased, water absorption and total porosity increased, but conversely, flexural and compressive strengths raised from 8.3 MPa and 19.3 MPa, respectively, for control cements to 11.3–11.8 MPa and 24.5–25.7 MPa for cements that incorporated 30–50 wt% SOFE. The increase in mechanical properties could be attributed to the synergistic formation of a hybrid N,C-A-S–H gel, resulting from the higher formation of N-A-S–H geopolymeric gel in comparison to C-A-S–H gel, owing to the higher amount of silica in the SOFE residue. The insulating capability of the cements improved as increasing amounts of SOFE residue were incorporated, with values ranging from 0.68 W/mK for the control binders to 0.34–0.15 W/mK for the cements that included 30–50% by weight of SOFE. The results of this study may help to promote the application of SOFE in the production of more environmentally friendly EAFS-based alkaline activation cements.

## Introduction

The development of alternative cementitious as substitutes for conventional cement has been gaining significant attention in the construction field in the last decades. The use of aluminosilicate-rich wastes, such as fly ash and slags for the replacement of Portland cement is limited typically ranging from 15 to 50 wt% of the total mass. This limitation is imposed due to the low hydration rate and higher drying shrinkage of the wastes, resulting in pozzolanic cements (Zhang 2011). The reactivity of these industrial by-products is enhanced in an alkaline environment, leading to the formation of alkali-activated materials (AAMs), a broad category that includes both concretes and pastes, commonly referred to as alkali-activated cements (AACs) when focusing specifically on the binder phase. Alkaline-activated cements are the most eco-efficient and promising green cements or hydraulic binder, due to their lower environmental impact. Therefore, they are materials that considerably reduce CO_2_ emissions without compromising economic costs (Khale and Chaudhar 2007). Two models of alkali-activated binding systems have been developed (Palomo et al. 1999). The first model involves activation of silicon- and calcium-rich raw materials such as electric arc steel slag, with an alkaline solution, resulting in the main reaction product being calcium aluminosilicate gel (C-A-S–H). In the second model of alkaline activation, silicon- and aluminum-rich precursors, such as metakaolin or F-class fly ash, are used with alkaline solutions, and the reaction products consist of three tridimensional Si–O-Al polymer chains. The second group, characterized by their polymeric structure, was named “Geopolymer” by Davidovits (1989).

The synthesis of alkali-activated cements is influenced by various factors, including the physicochemical characteristics of the precursor, the type and quantity of activator employed, and the curing conditions (Collins and Sanjayan 1999, 2000; Bakharev et al. 1999; Bernal et al. 2010). Consequently, it can be inferred that parameters like workability, mechanical strength, microstructure, or durability of the produced materials can be modified by varying the physicochemical properties of the industrial wastes used as precursors (Part et al. 2015).

Electric arc furnace slag (EAFS) are slags obtained as a by-product in steel production, when molten steel is separated from impurities (Khan et al. 2016). For every ton of steel produced, 0.13 tons of EAFS are generated. The amount of EAFS produced annually in Spain is 1,200,000 tons (MITECO, 2021). In Europe, EAFS account for 42.9% of total steel waste (Worldsteel Association 2021). Regarding their chemical composition, they are mainly composed of CaO, SiO_2_ and Fe_2_O_3_, with small amounts of Al_2_O_3_ (Oyebisi et al. 2020). This industrial by-product has wide applications in civil and construction materials, especially as aggregate for road construction and soil improvement (Worldsteel Association 2021; Nguyen et al. 2022; Sukmak et al. 2023) and as aggregate for the manufacture of concrete (Rojas et al. 2023). In addition, due to its chemical composition, EAFS has potential as a replacement material for Portland cement (Kim et al. 2015) and for the manufacture of alkaline activation cements (Mishra and Lahoti 2023; Ozturk et al. 2019; Gómez-Casero et al 2022).

Spent oil filtering earths (SOFE) are industrial wastes obtained from oil and grease filtration processes. Filtering earths are typically diatomaceous earths with small particle sizes and large specific surface areas. They contain low levels of crystalline silica and high amounts of amorphous silica. These materials often retain 20–40 wt% of residual oil, metallic impurities, and various organic compounds (Boey et al. 2011). Spent filtering earths pose challenges in terms of handling and management due to their composition, volume generated, and the limited viable options for their recovery. As a result, the most practical current solution primarily involves the collection and proper disposal of this waste by authorized agents.

Partial replacement of slags by silicon-rich wastes in raw material has been previously studied by other authors. Chi and Huang (2013) studied the properties of alkali activated mortars made from fly ash and slag combined in different proportions and found that 50–50 wt% mixtures led to optimum results when 6% Na_2_O concentration was used in the activator. Main hydration products are alkali amorphous aluminosilicate and low-crystallinity calcium silicate hydrate gel. Puertas et al. (2000) studied the mechanical strength and hydration products of fly ash/slag materials activated with sodium hydroxide, concluding that best results were found when 50–50 wt% mixtures were activated with 10 M NaOH solution and cured at 25 °C. Fly ash–slag proportion turned out to be one of the main factors influencing the mechanical properties development. Mishra et al. (2023) studied the effects of EAFS and fly ash mix design parameters. Fly ash, at 0–75 wt%, partially replaced EAFS in the composite binary mix system. The optimum mixture (compression strength of ~ 39 MPa) was obtained for a 75–25 wt% composition of EAFS and fly ash activated with a sodium silicate/sodium hydroxide solution (12 M) in a 2:1 ratio, due to the coexistence of C-A-S–H and N-A-S–H gels. Mehta and Siddique (2018) studied the influence of the use of rice husk ash (RHA) as partial substitution of ground granulated blast furnace slag (GGBS) for the development of sustainable geopolymer concrete. Their findings showed that denser microstructure and best mechanical properties for the geopolymeric concrete manufactured were obtained with the addition of 15% of RHA owing to the coexistence of polymerization products (NASH gel) and calcium-based hydration products (CSH and CASH). Liang and Yao (2023) investigated the effect of diatomite incorporation on the early compressive strength (72 h) of alkali activated slag pastes. The pastes containing diatomite showed a delay in the reaction process compared to the reference paste, thus the compressive strength of the pastes after 3 days of curing decreases with the incorporation of diatomite. However, the effect of the incorporation of oil filtering earths (spent diatomite) to EAFS-based alkaline activation pastes is unknown, as it has not been previously studied.

The present work investigated the effect of the addition of different amounts of spent oil filtering earths (SOFE) to alkaline-activated cements based on electric arc steel slag (EAFS) cured at ambient temperature. The effect of the mixture composition on the physical, mechanical, and thermal properties was evaluated as a function of the curing time (7 and 28 days). The chemical, mineralogical, and microstructural composition of the pastes was studied by various characterization techniques: FTIR, XRD, and SEM–EDS. A study of the immobilization of heavy metals present in wastes has been performed by leaching test. Therefore, this work provides new insights into the properties of SOFE-EAFS blended cements.

## Experimental program

### Raw materials

**Fig. 1:**
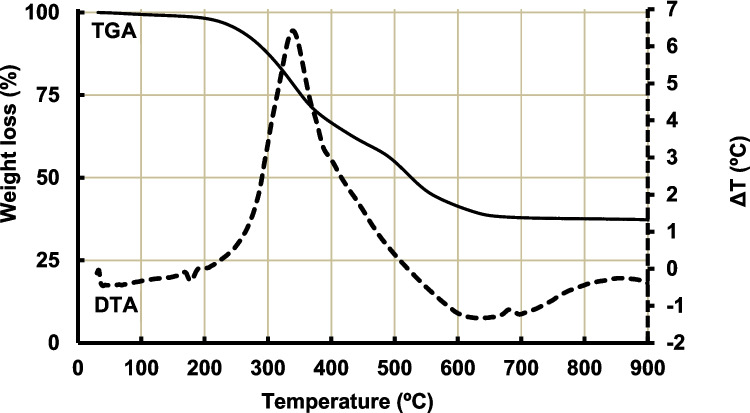
TGA-DTA study of spent oil filtering earths (SOFE)

The electric arc furnace slag (EAFS) was sourced from “Siderúrgica Sevillana” company situated in Alcalá de Guadaira, Seville (Spain) and “Siderúrgica Balboa” company located in Jerez de los Caballeros, Badajoz (Spain). The slag, as received, presents a particle size of 20–40 mm. This subproduct was crushed in a Retsch model BB100 jaw crusher to a particle size below 4 mm and then ground in an Orto Alresa ML007 ball mill and sieved to a particle size of less than 0.100 mm. The spent oil filtering earths (SOFE) were supplied by Aceites del Sur Coosur, S.A, a company located in Vilches, Jaén (Spain). The SOFE residue underwent a heat treatment process to eliminate the organic matter. To ascertain the suitable temperature for this treatment, a thermogravimetric-differential thermal analyses (TGA-DTA) was performed on the dry residue. A Mettler Toledo Thermal Analyzer model was utilized for this purpose. The airflow was maintained at 50 mL/min, and the heating rate was set at 10 °C/min, scanning from 30 to 900 °C. TGA-DTA curve of SOFE (Fig. 1) showed a mass loss of 1.0% between 30 and 150 °C associated with the evaporation of physically adsorbed water and/or mechanically trapped water, which is associated with a small endothermic event in the DTA curve around 100 °C (Mendioroz et al. 1989). Between 150 and 600 °C, the combustion of organic matter (57.7%) takes place, as shown the two exothermic peaks centered at 345 °C and 400 °C which correspond to the sequential combustion of different components: the first one, more intense peak is attributed to the burning of volatile compounds or oils, while the second one, less pronounced peak is associated with the degradation of non-volatile components (Mendioroz et al. 1989). Possible effects related to recrystallization or transformation of amorphous silica to crystalline phases such as cristobalite can occur at higher temperatures as indicated by the small exothermic effect centered at 685 °C. It was ascribed mainly to the de-hydroxylation of Si − OH groups. The temperature for the heat treatment was fixed at 700 °C (2 h, heating rate of 10 °C/min), since the mass remains constant from this temperature onwards. The calcined residue was homogenized in a ball mill and sieved to a particle size of less than 0.100 mm. The Blaine surface area of the EAFS and SOFE residues was 3090 and 3215 m^2^/g and the specific gravity was 3.63 and 2.34, respectively (Table 1).

**Table 1:** Physical properties and particle size distribution of raw materials

Raw materials	Blaine surface (m^2^/g)	Specific gravity	*D*_10_ (µm)	*D*_50_ (µm)	*D*_90_ (µm)
EAFS	3090	3.63	2.9	24.0	78.4
SOFE	3215	2.34	5.56	22.5	61.9

**Fig. 2:**
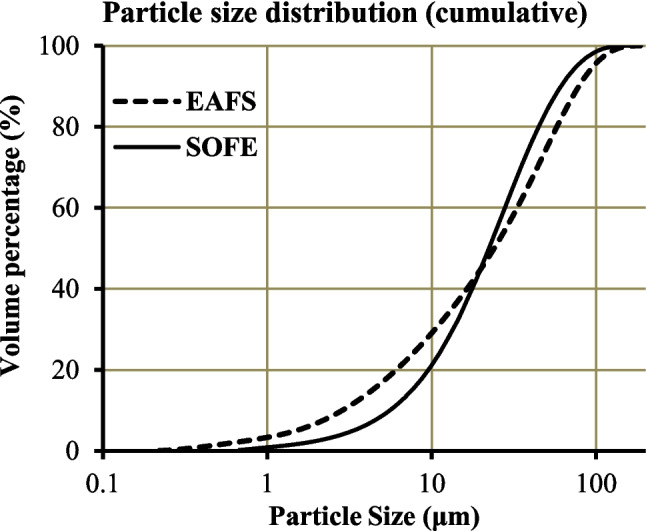
Cumulative particle size distributions of EAFS sand SOFE

Physical properties and particle size distributions of the EAFS and SOFE are provided in Table 1 and illustrated in Fig. 2, respectively.

The chemical composition of the subproduct EAFS and SOFE residue was analyzed using X-ray fluorescence (XRF), as shown in Table 2, employing a Zetium Malvern Panalytical instrument.

**Table 2:** Chemical composition of electric arc furnace slag (EAFS) and spent oil filtering earths (SOFE)

Raw material	SiO_2 _(%)	Al_2_O_3 _(%)	Fe_2_O_3 _(%)	CaO (%)	MgO (%)	MnO (%)	Na_2_O (%)	K_2_O (%)	TiO_2 _(%)	P_2_O_5 _(%)	SO_3 _(%)	LOI (%)
EAFS	18.36	10.94	26.07	28.37	6.53	4.82	0.14	0.02	1.01	0.31	0.26	1.95
SOFE	84.30	5.87	2.41	0.96	0.38	0.02	3.47	1.50	0.57	0.33	0.05	0.14

The EAFS is primarily composed of CaO (28.4%), Fe_2_O3 (26.1%), SiO_2_ (18.4%), and Al_2_O_3_ (10.9%), with lesser amounts of MgO (6.5%) and MnO (4.8%). On the other hand, SOFE is predominantly composed of SiO_2_ (84.3%) and contains smaller proportions of Al_2_O_3_ (5.9%), Fe_2_O_3_ (2.4%), and K_2_O (1.5%). SOFE represents a promising source of silica for use in the production of alkali-activated cements.

**Fig. 3:**
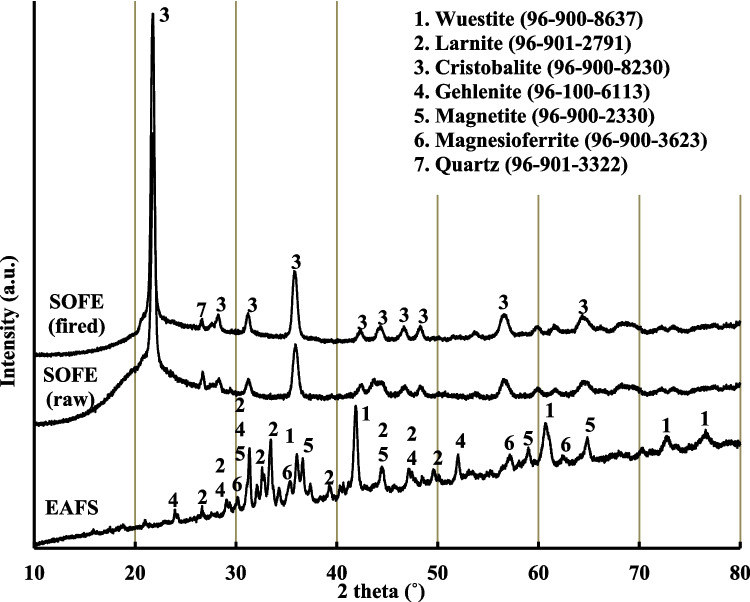
XRD patterns of EAFS and SOFE as raw material and after thermal treatment

X-ray diffraction (XRD) examinations were carried out, with measurements ranging from 10 to 80° at 2*θ* degrees using Cu-Kα radiation (*λ* = 1.54 Å), to probe the mineralogical composition of the precursors. HighScore software was used for the determination of crystalline phases. The XRD analysis revealed that EAFS primarily consists of larnite (Ref. cod. 96–901–2791), gehlenite (Ref. cod. 96–900–6113), and wüstite (Ref. cod. 96–900–8637) as the predominant crystal phases. Additionally, lower amounts of magnetite (Ref cod. 96–900–2330) and magnesioferrite (Ref. cod. 96–900–3523) were also detected. In contrast, SOFE both in its raw and fired states were chiefly characterized by the presence of cristobalite (Ref. cod. 96–900–8230). This phase is even more prominent after the calcination process applied to this raw material, as evidenced by the sharpening of the cristobalite related peaks, and the increase of their intensities. l. Additionally, quartz (Ref. cod. 96–901–3322) was also detected in SOFE, as a very minor component (Fig. 3).

**Fig. 4:**
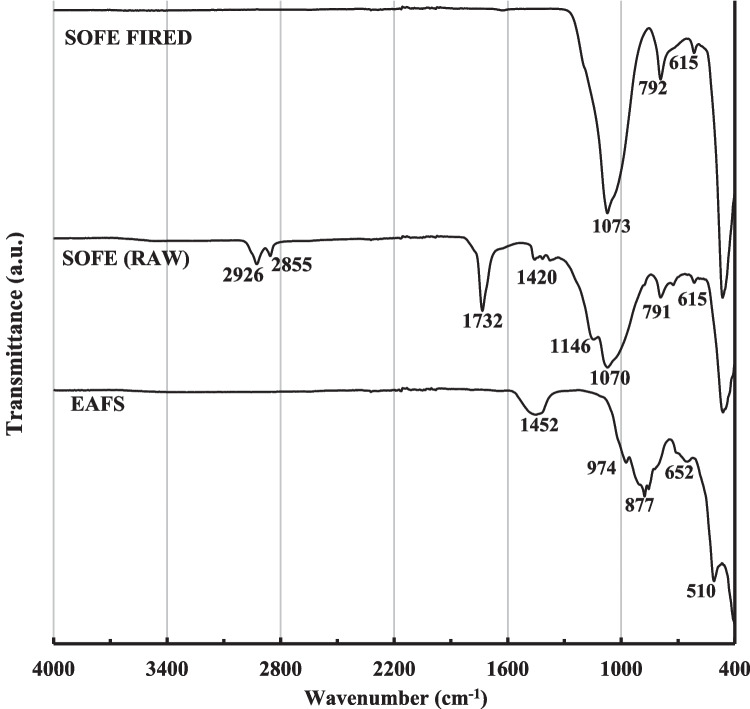
FTIR analysis of EAFS and SOFE as raw material and after thermal treatment

**Fig. 5:**
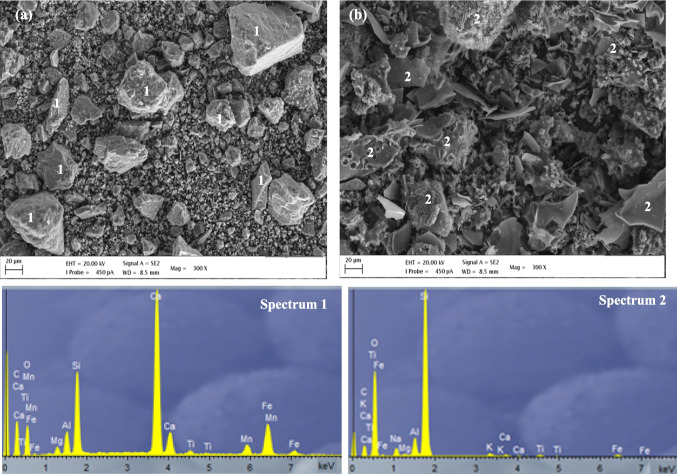
SEM–EDS image of raw materials: **a** EAFS and **b** SOFE

Raw materials were also analyzed by attenuated total reflectance Fourier transform infrared spectroscopy (ATR-FTIR) in the range of 4000 to 400 cm^−1^, using a Vertex 70 Bruker instrument, and the results are shown in Fig. 4. The main bands of the EAFS precursor centered at 1452, 974, and 652 cm^−1^ are attributed to stretching vibrations of the Al–O–Si and Si–O-T bonds (Walkley et al. 2016; Morrow and McFarlan 1992; Felaous et al. 2023). The band centered at 510 cm^−1^ is due to the bending modes of the O-T-O bonds of the TO_4_ tetrahedral (T = Si or Al). The band located at 877 cm^−1^ is attributed to the stretching vibration of the Fe–O (Balaguera and Botero 2020). The SOFE spectrum shows bands centered at 1073 cm^−1^ and 792 cm^−1^ associated mainly with the asymmetric and symmetric stretching vibration of the Si–O-Si (Al) bonds, respectively. The band centered at 464 cm^−1^ is related to the bending vibration of the Si–O-Si (Al) bond (Hassan et al. 2019). The small band appearing at 615 cm^−1^ is attributed to the vibration of the Al-O bond (Zhao et al. 2020). These peaks were also present in the raw material before calcination. On the other hand, the peaks located around 2900 and 1400 cm^−1^, which correspond to the stretching and bending vibrations of C–H bonds in CH_x_ groups, respectively, are attributed to the organic fraction present in the residue and disappear after the thermal treatment (Amaral et al. 2022). The same applies to the peak at 1732 cm^−1^, associated with the stretching vibration of the C = O double bond found and the in organic structures and the bands centered at about 1420 cm^−1^ associated with the asymmetric bending vibration of the carbonate ion (CO₃^2^-) (Liu et al. 2025). The morphology of the EAFS and SOFE particles was observed by scanning electron microscope (SEM) (Figure 5) using a Carl Zeiss model Merlin assisted by Energy Dispersive X-ray Spectroscopy (EDS). The samples were mounted on an aluminium grid and subsequently coated with carbon using the JEOL JFC 1100 sputter coater. It can be seen that both precursors present particles in a wide range of sizes. The EAFS presents an irregular shape, with a predominance of angular particles. EDS analysis showed the presence of calcium, iron, silica, aluminum magnesium and manganese (Spectrum 1). In the SOFE residue different shapes can be identified with predominance of a rough surface texture with porous microstructure which is the reason why it is used as a filter. In this case, EDS analysis indicated a predominance of silica with a lower content of aluminum and other components such as sodium, potassium, iron and calcium (Spectrum 2). These EDS results agree with data presented in Table 1.

### Test plan

The alkaline activating solution composed of 50 wt% NaOH 8 M and 50 wt% of a commercial solution of sodium silicate (Na_2_SiO_3_) was prepared by dissolving solid sodium hydroxide (NaOH) (98% purity, Panreac) in water and the subsequent addition of the necessary amount of Na_2_SiO_3_ solution (29.2% SiO_2_; 8.9% Na_2_O; and 61.9% H_2_O, Panreac). The silica modulus of the activating solution (Ms = mol SiO_2_/molNa_2_O) was equal to 0.95. The alkaline solution was prepared 24 h before use.

SOFE was incorporated as a partial substitute for EAFS in the raw material of alkali-activated binders. This was done to investigate how the replacement percentage affects the technological properties of alkali-activated slag cements. The EAFS residue was replaced by spent oil filtering earths in the range of 0–50 wt%, with increments of 10 wt%. Additionally, a composition consisting of 100 wt% slag was prepared as a reference. Specific information regarding the mixture proportions can be found in Table 3. The samples were designated as EAFS-xSOFE, with “x” representing the percentage of SOFE used to replace EAFS.

**Table 3:** Mix formulations of alkali activated cement in weight proportion and flow test data

Nomenclature	EAFS (g)	SOFE (g)	Relation liquid/binder (L/b)	Activating solution (g)	Diameter flow test (mm)
EAFS- 0SOFE	300	0	0.30	90	76.17 ± 3.05
EAFS- 10SOFE	270	30	0.45	135	74.13 ± 1.58
EAFS- 20SOFE	240	60	0.55	165	73.16 ± 2.46
EAFS- 30SOFE	210	90	0.65	195	73.24 ± 0.48
EAFS- 40SOFE	180	120	0.75	225	72.90 ± 1.22
EAFS- 50SOFE	150	150	0.90	270	72.12 ± 0.78

The quantity of activating solution added to the mixture was increased as the percentage of SOFE rises to ensure a consistent workability for all the pastes.

The flow diameter was maintained within a ± 5% variation range to guarantee consistency in paste handling and molding. The consistency of the pastes was determined by performing a flow test in accordance with ASTM C- 1437:20 (ASTM C- 1437, 2020). This method allows for an indirect assessment of the plasticity and fluidity of the mixtures. Table 3 shows the results of the workability of fresh pastes. The incorporation of SOFE generates a progressive reduction in the flowability of the pastes. The average value decreases from 76.2 mm in the control cements (EAFS- 0SOFE) to 72.1 mm in the cements incorporating 50 wt% SOFE (EAFS- 50SOFE). SOFE required a higher volume of solution to achieve a paste-like consistency due to its higher specific surface area and microporosity (Dong et al. 2022). The decrease in slump values with the addition of other silica-rich sources such as silica fume to ferrosilicon slag was also observed by Jena y Ramakanta (2021). Cheng et al. (2024) employed a water-reducing agent in the synthesis of alkali-activated mortars based on bast-furnace slag (BFS) with diatomaceous earth (DE) (10–30 wt%) substitution. Due to the numerous internal pores of DE and its high water absorption, the incorporation of DE increases the water demand of the alkali-activated BFS-DE mortar, resulting in a significant decrease in flowability. Throughout the curing period, no significant volumetric instabilities such as expansion or shrinkage cracking were observed, confirming the dimensional stability of the formulations. However, the higher liquid/binder ratio required for SOFE-containing pastes may have influenced the early strength development and CO_2_ emissions.

Regarding the sustainability of SOFE-EAFS-based alkali-activated cements, it is well established in the production of alkali-activated materials that the use of commercial alkaline activators represents a significant share of the Global Warming Potential (GWP) of the final product, often exceeding 50–60% depending on the formulation considered (Salas et al. 2018; Mir et al. 2022). Specifically, the Ecoinvent Dataset 3.6, widely used for life cycle assessment (LCA) studies, provides the carbon footprint of various components (Table 4).

**Table 4:** Inventory values for commercial alkali activators

	Description	GWP [kg CO_2_ eq/Kg]
NaOH	Sodium hydroxide, without water, in 50% solution state {RER}| chlor-alkali electrolysis, membrane cell | APOS, U	0.824
Na_2_SiO_3_	Sodium silicate, solid {RER}| market for sodium silicate, solid | APOS, U	0.759

Based on this dataset, the preparation of 1 kg of the alkali solution used in this study could result in an estimated GWP of approximately 0.246 kg CO₂-eq, excluding water consumption and mixing operations. As a result, the proposed substitution of EAFS with SOFE is associated with an increase in GWP. In addition, the preparation of SOFE requires heat treatment at 700 °C, which implies additional energy consumption. However, a comprehensive LCA is beyond the scope of this study, as it would require defining a specific production site to account for the transportation distances of both raw materials. Additionally, the potentially lower energy demand for SOFE grinding could contribute to identifying a viable incorporation rate in future studies.

### Sample preparation

**Fig. 6:**
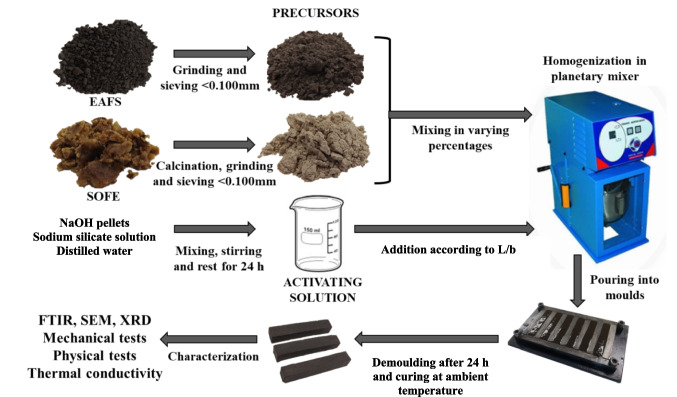
Experimental plan

During the initial phase, EAFS and SOFE were mixed in a planetary mixer for 90 s. Subsequently, the activating solution was introduced, and the entire mixture was homogenized for additional 90 s. At this point, the paste was poured into steel molds to create prismatic samples measuring 60 × 10 × 10 mm and cylindrical molds with dimensions of 55 mm in diameter and 15 mm in height to create specific samples for thermal conductivity measurement. The molds containing the paste were subjected to 60 strokes on a punching table and then covered with plastic film for the first 24 h to avoid evaporation and cured at ambient temperature. Following this, the samples were removed from the molds and stored under laboratory conditions (23 °C and 60% relative humidity) until the test ages of 7 and 28 days. The experimental plan’s schematic is illustrated in Fig. 6.

### Test methods

The measurements for bulk density and water absorption of the binders were conducted in accordance with UNE-EN 1015–10:2020 standard (EN 1015–10, 2020). Real density was determined through pycnometry, using ethanol as the displacing fluid. Total porosity was calculated by taking the ratio of bulk density to real density. The flexural and compressive strengths of alkali-activated cements were determined following the guidelines of UNE-EN 1015–11:2000/A1:2007 (EN 1015–11, 2000). For flexural strength testing, MTS Insight 5 machine with a capacity of 5 kN and a displacement rate of 0.2 mm/min was employed. Compressive strength testing utilized a universal testing machine, specifically the MTS 8101 with a capacity of 100 kN and a displacement rate of 2 mm/min. The measure of thermal conductivity for the binders at 20 °C was carried out using a FOX 50 TA Instruments Thermal Flow meter, as per the ISO 8302:1991 standard (ISO 8302 1991).

The powdered alkaline-activated cements were characterized by X-ray diffraction (XRD) and the fracture surface of the binders after the compressive strength test was analyzed by SEM–EDS using the same equipment and conditions as for the precursors. The powder samples were analyzed by attenuated total reflectance Fourier transform infrared spectroscopy (ATR-FTIR) using the same equipment and conditions as for raw materials.

The pH of alkaline activation cements was evaluated as a function of time by dissolving 1 g of sample in 10 g of deionized water at room temperature (∼25 °C). The mixture was stirred to ensure homogeneous distribution prior to measurement. The pH was determined using a Crison 25 + pH meter.

Leaching tests were carried out following an adaptation of the European standard EN 12457–1:2003 (EN 12451–1, 2003). For this purpose, 80 g of the cured cements were crushed for 28 days until a particle size of less than 4 mm was obtained. The samples were mixed with distilled water at a liquid/solid ratio of 2 (160 g of water) and subjected to agitation at 10 rpm for 24 h. Subsequently, the suspensions were allowed to settle and the supernatant was filtered using a cellulose nitrate filter with a pore size of 0.6 µm to obtain the liquid extract. The resulting leachates were analyzed by inductively coupled plasma mass spectrometry (ICP-MS) using an ICP-MS spectrophotometer (Agilent, model 7900).

## Results and discussion

### FTIR data

**Fig. 7:**
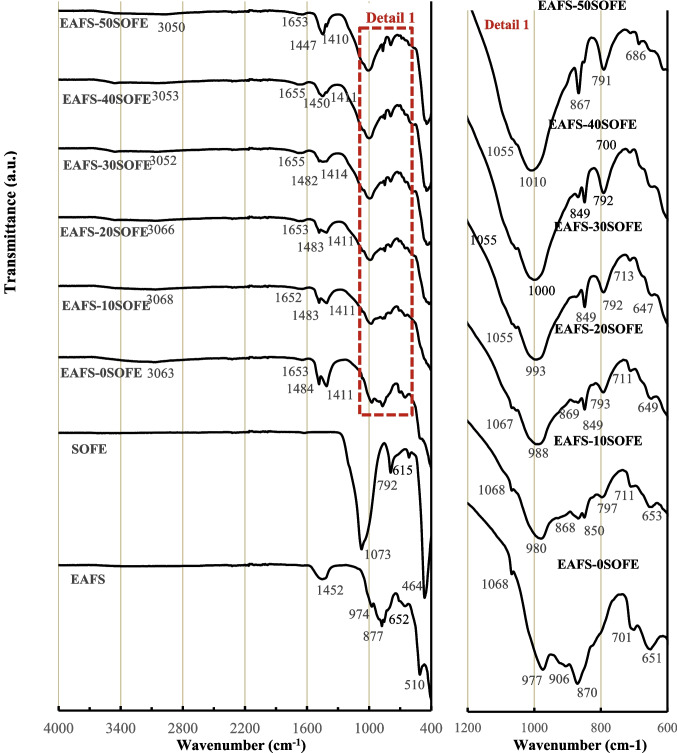
FTIR spectra of alkaline-activated cements and raw materials (left) and detail of bands of interest (right)

Figure 7 shows the FTIR spectra of the alkaline-activated cements after 28 days of curing. For comparison, the spectra of the EAFS and SOFE raw materials are included. The spectrum of the EAFS- 0SOFE control cement shows a band centered at 977 cm^−1^ associated with the stretching vibrations of the Si–O bond in the SiO_4_ tetrahedral composing the calcium aluminate gel (C-A-S–H) (García-Lodeiro et al. 2011). A slight shoulder centered at 1068 cm^−1^ associated with the formation of N-A-S–H geopolymeric gel is also observed (García-Lodeiro et al. 2011). In addition, the band attributed to the bending modes of the O-T-O bond of the TO_4_ tetrahedral centered at 495 cm^−1^ shows a decrease and a shift to a lower wave number with respect to the precursor, indicating the dissolution of the slag and the formation during the alkaline activation process of C-A-S–H gel (Nikolić et al. 2020).

The substitution of the slag by SOFE produced a shift of the band attributed to the C-A-S–H gel, at higher wavelengths up to 1010 cm^−1^ with the incorporation of 50 wt% of residue which could indicate the incorporation of sodium ions in the -Si–O- bonds confirming the presence of hybrid (C,N)-A-S–H gel. That is, SOFE participates in geopolymerization and affected the molecular structure of the formed gels, probably decreasing the Ca/Si ratio of the C(N)-A-S–H structures and promoting the formation of N(C)-A-S–H structure (Puligilla and Mondal 2015). Therefore, SOFE incorporation correlates with increased N-A-S–H geopolymer gel formation (Chen et al. 2018). In addition, this main band is wider, which could indicate the coexistence of several types of gels such as (N,C)-(A)-S–H due to the displacement of sodium by calcium (Somna et al. 2011). Shifting to a higher wavenumber with the incorporation of increasing amounts of SOFE implies less Al incorporation into the geopolymer network (Nikolov et al. 2020). That is, higher SOFE incorporation caused fewer Si–O-Al bonds to form, which are weaker than Si–O-Si bonds, which could account for the increase in compressive strength. On the other hand, the intensity of this band increases as the SOFE content increases. This could be due to the lower incorporation of Ca in the Si–O-Si and Si–O-Al bonds forming a larger amount of N-A-S–H gel that coexists with the C-A-S–H matrix, as indicated above (Hui-Teng et al. 2021).

In all the spectra of the alkaline-activated cements, bands located at approximately 3050–3068 cm^−1^ and 1652–1655 cm^−1^ assigned to the stretching and bending vibrations of the O–H bonds, respectively, are observed (Catauro et al. 2014). These bands confirm the presence of water in the products due to water molecules adsorbed on the surface or trapped in the pores of the reaction products during the alkaline activation process (Tho-In et al. 2018). The bands centered around 1450 and 870 cm^−1^ are related to the vibration of the O-C-O bond of CO_3_^2−^. The band at 1450 cm^−1^ presents a split into two bands centered at approximately 1483 and 1411 cm^−1^ for cements incorporating up to 30 wt% SOFE and between 1450 and 1410 cm^−1^ for binders incorporating 40 and 50 wt% of the residue. The splitting of these bands is due to the interaction of the carbonate ions with different metal ions, such as Na^+^ and Ca^2+^. The highest energy band corresponds to calcium ions which is the divalent cation (Fine and Stolper 1986). While the CO_3_^2−^ bound sodium ions are assigned to the bands centered around 1450 cm^−1^. The band observed at 1411 cm^−1^ can be attributed to C-O bond vibrations in the CO_3_^2−^ ions with no interaction with the metal ions or with a weak interaction (Fine and Stolper 1986).

### XRD data

**Fig. 8:**
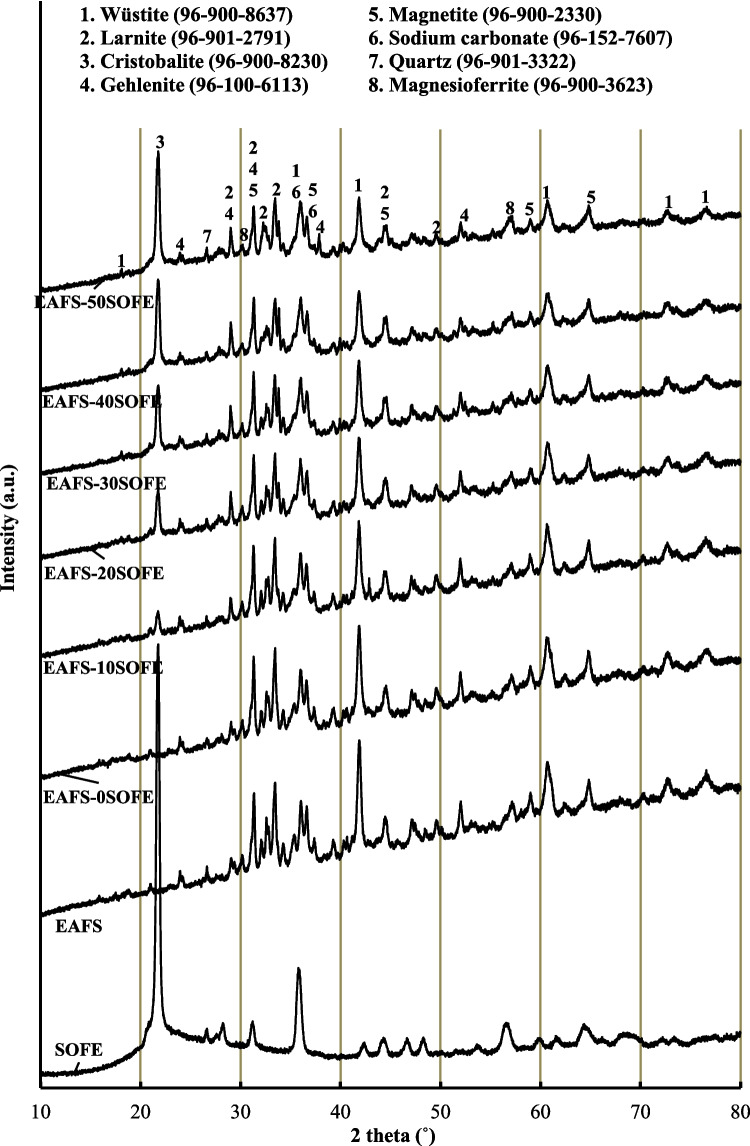
XRD pattern of EAFS-SOFE alkaline-activated cements and comparison with raw materials

The results of the XRD patterns of the alkaline-activated cements after 28 days of curing are presented in Fig. 8. It is observed that crystalline phases present in the EAFS such as larnite, gehlenite, wüstite, and magnetite are present in all alkaline-activated cements after reaction with the activating solution. As the amount of SOFE increases, it is observed that the intensity of the peaks corresponding to the crystalline phases of the slag decreases, while an increase of the peak intensity corresponding to the cristobalite present as crystalline phase of the SOFE residue increases. This fact suggests the difficulty of dissolving the crystalline phases present in the precursors under the alkaline activation conditions employed. The presence of crystalline starting phases in alkaline-activated cements is consistent with previous studies using EAFS as a precursor (Ozturk et al. 2019; Gómez-Casero et al. 2022) and diatomaceous earths (Coelho et al. 2023; Liang and Yao 2023). In addition, the appearance of a new crystalline phase identified by XRD as sodium carbonate was also observed according to the FTIR data. Excess sodium from the activator reacts with atmospheric CO_2_ to form this phase. The halo attributed to the glassy phase in the EAFS and SOFE feedstocks shifts slightly towards higher 2*θ* values (2*θ* = 20–40°) and is associated with the amorphous phase. This modification is indicative of the formation of an alkaline aluminosilicate gel (N-A-S–H gel). This halo increases slightly with the incorporation of increasing amounts of SOFE which could indicate a higher formation of the N-A-S–H reaction product with respect to the predominant C(N)-A-S–H gel when used as EAFS precursor according to SEM data (Hui-Teng et al. 2021).

### SEM–EDS analysis

**Fig. 9:**
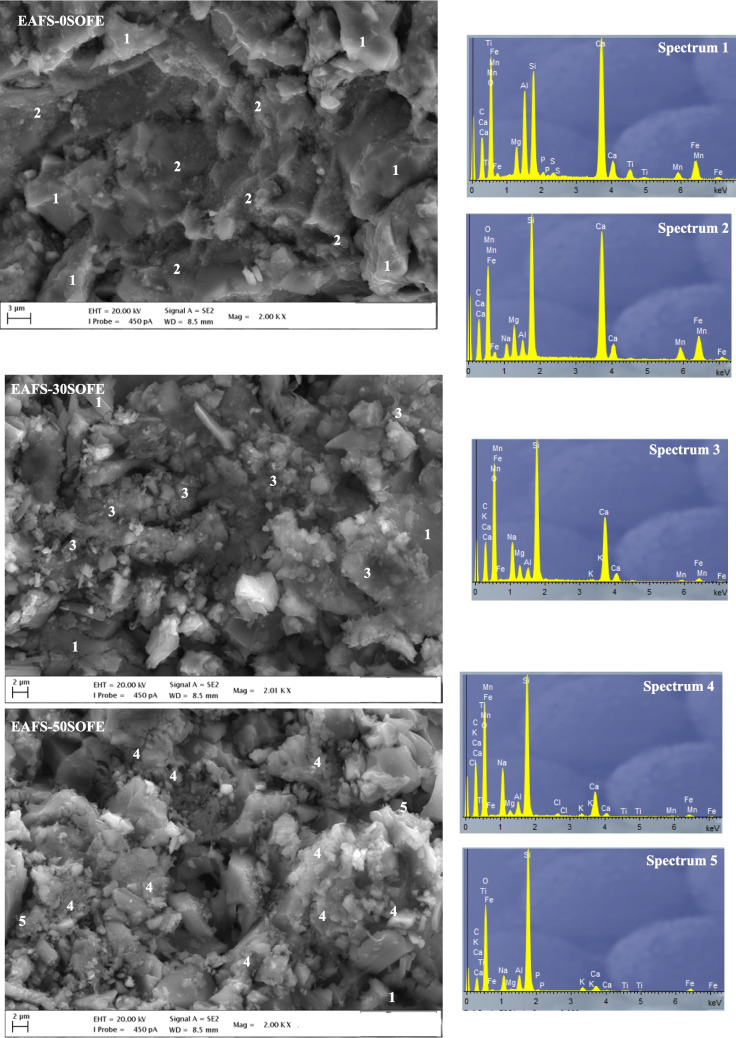
SEM micrographs and EDS spectra analysis of **a** EAFS- 0SOFE, **b** EAFS- 30SOFE, and **c** EAFS- 50SOFE alkaline-activated cements at 2kx magnification

**Fig. 10:**
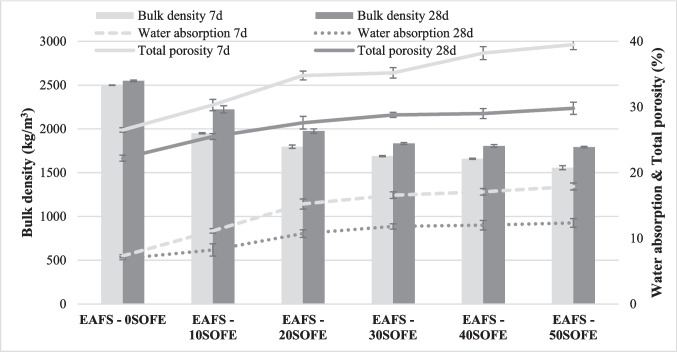
Bulk density, water absorption and total porosity of EAFS-xSOFE alkaline-activated cements at 7 and 28 days of curing

Figure 9a–c shows selected SEM images of alkaline-activated binders containing 0 wt%, 30 wt%, and 50 wt% of SOFE after 28 days of curing. It can be seen that the replacement of EAFS by SOFE in the alkaline-activated cements results in less dense structures, with higher pore content and lower number of unreacted particles. The EDS spectra of points 1 indicate that the EAFS- 0SOFE control sample and the EAFS- 30SOFE cement contain some unreacted or partially reacted slag particles. The EDS spectra of point 2 in the control cement suggests the formation of calcium aluminosilicate hydrate gel (C-A-S–H) with small amounts of sodium (N)-C-A-S–H. Therefore, EAFS contains a high proportion of calcium which favors the formation of C-A-S–H as the main reaction product (Bouaissi et al. 2019). The EAFS- 30SOFE and EAFS- 50SOFE samples have a different microstructure (Fig. 9b, c), which is consistent with the changes in compressive strength (see Fig. 10). The incorporation of 30 and 50 wt% SOFE leads to the active participation of SOFE silicon to produce more sodium-rich gels during the geopolymerization process, suggesting that the main hydration product was (N,C)-A-S–H gel with variable composition, richer in Na, as indicated by points 3 and 4 of the EDS spectra. Thus, in the EAFS-SOFE-based geopolymers, a higher compressive strength is produced compared to the 100% EAFS control cement (Phoo-Ngernkham et al. 2017; Rashad et al. 2021). Additionally, some unreacted SOFE particles are observed in EAFS- 50SOFE cement (point 5).

### Bulk density, water absorption, and total porosity

Figure 10 shows the results of bulk density, water absorption, and total porosity for each type of binder according to the replacement percentage of EAFS with SOFE at 7 and 28 days of curing. Water absorption and total porosity values follow a similar tendency and contrary to the bulk density variation. Reference sample (EAFS- 0SOFE) bulk density was determined to be 2499 and 2548 kg/m^3^ while water absorption values observed were 7.3 and 6.9%, with a total porosity of 26.5 and 22.2% for 7 and 28 days, respectively. Unreacted EAFS particles act as microfillers for pores, voids, and empty spaces (Bernal et al. 2011) increasing bulk density and decreasing water absorption and total porosity. The incorporation of increasing amounts of SOFE resulted in a gradual decrease in bulk density, accompanied by a corresponding growth in water absorption and total porosity. Tests conducted at 7 and 28 days revealed that the lowest bulk density values (1549 and 1792 kg/m^3^) and the highest water absorption (17.9 and 12.4%) and total porosity percentages (39.5 and 29.8%) were observed in the EAFS- 50SOFE sample. The variation in bulk density could be due to the lower real density of the SOFE residue (2348 kg/m^3^) compared to EAFS (3628 kg/m^3^) which contributed to a lower particle mass/volume ratio as its weight percentage increased (Nikolić et al. 2016). On the other hand, water absorption and total porosity increase and bulk density decreases with increasing liquid/binder ratio (Table 2), being higher as increasing amounts of SOFE are incorporated to maintain the workability of the paste. The subsequent elimination of the water used due to the evaporation process during curing led to higher porosity especially at low hydration times, 7 days. The higher amount of SiO_2_ delayed the reaction of Si and Al ions and produced a lower density geopolymer gel resulting in lower mechanical properties at low cure times (Tho-In et al. 2018). SOFE pastes have shown to have a lower degree of reaction at short hydration times due to a delayed reaction process thus reducing the amount of reaction products at low curing times (Liang and Yao 2023). The decrease in bulk density at low curing times, 7 days with the addition to slags of other silica-rich sources such as RHA and the increase in bulk density after 28 days of curing has also been observed by other authors (Sabbar Abbas et al. 2023). However, the notable decrease in porosity observed after 28 days of curing probably due to the still ongoing development of the geopolymerization processes associated to the higher reactivity of the binary mixtures that would produce a bigger amount of hybrid geopolymer gel (N,C-A-S–H) in accordance with to SEM analysis and FTIR spectra. The hybrid gel formed at longer hydration times is able to fill the porous product formed by water evaporation, which helps to reduce the amount of water absorption and total porosity. Therefore, bulk density, water absorption, and total porosity are significantly affected by hydration time. As the curing time increases, bulk density is observed to increase while there is a reduction in water absorption and total porosity. These findings are consistent with the evolution of the geopolymerization and activation processes which, after 28 days of curing gave rise to more compact materials, reducing the porous structure (Hu et al. 2019). The inclusion of other silica-rich sources such as silica fume produced an increase in bulk density and a decrease in water absorption after 28 days of curing by decreasing the voids in the geopolymer matrix through its packing and filling effect (Jena and Panigrahi 2021).

### Flexural and compressive strength

**Fig. 11:**
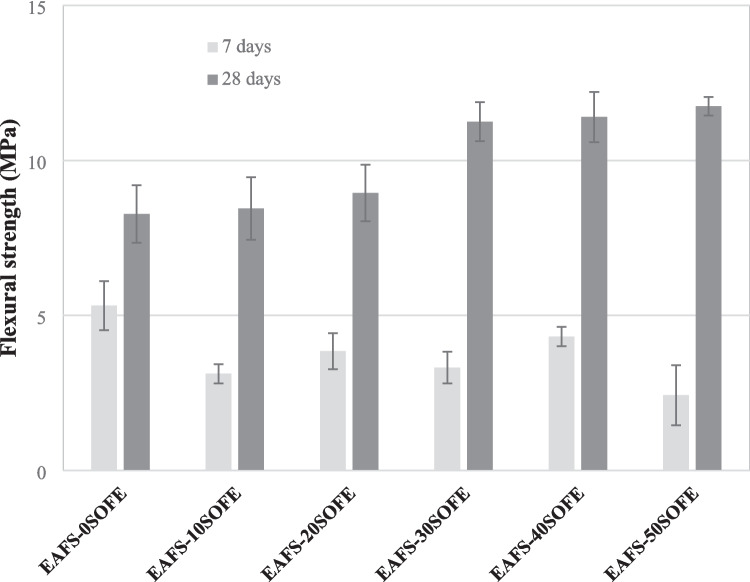

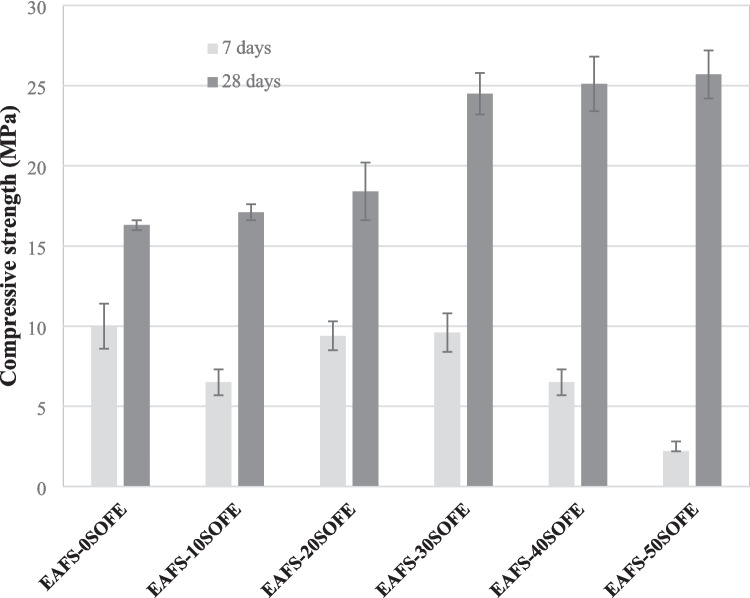
Mechanical properties (flexural and compressive strength) of alkali activated cements as function of SOFE content at 7 and 28 days of curing

Figure 11 shows the results of flexural and compressive strength of every binder according to the replacement percentage of EAFS with SOFE at 7 and 28 days of curing. As can be seen, the flexural and compressive strength are affected by the proportion of SOFE and hydration time. The flexural and compressive strengths of the EAFS- 0SOFE control cements after 28 days of curing are the lowest at 5.3 and 16.3 MPa respectively despite the fact that these alkaline-activated cements have higher bulk densities and lower total porosity. These results may be due to inadequate reactive silica resulting in an insufficient amount of calcium aluminosilicate gel formation, (N),C-A-S–H (Ozturk et al. 2019). These results are comparable with those obtained for EAFS-based cements by other authors when EAFS are activated with an 8 M NaOH solution cured at 80 °C (Mishra and Lahoti 2023). The substitution of EAFS by SOFE produced an increase in flexural and compressive strength after 28 days of curing, being this increase more noticeable with the addition of 30–50 wt% SOFE, obtaining increases of 36.0–42.1% and 50.3–57.7% respectively with respect to the control cement. The positive effect of SOFE on strength, since the specimens have lower density and higher total porosity, is mainly due to the reactive SiO_2_ containing (Yilmaz and Ediz 2008). Silicon and aluminum from SOFE and at the same time, silicon, aluminum, and calcium from EAFS are dissolved in an alkaline medium.

The presence of dissolved calcium will react with the soluble silicate and precipitate as a geopolymer gel formed by the presence of SOFE. The alkaline activation of the calcium-rich EAFS in the presence of a silica source (SOFE) causes the two reactions to occur simultaneously, formation of C-A-S–H and N-A-S–H gel. The amount, nature, and size of the hydration products present will determine the mechanical properties of the resulting matrices (Yip and van Deventer 2003). The coexistence of C-A-S–H gel (from EAFS) and N-A-S–H gel (from SOFE) gives rise as observed by SEM and FTIR studies to a hybrid (N,C)-A-S–H gel richer in Na as increasing amounts of SOFE are incorporated. The simultaneous formation of N-A-S–H and C-A-S–H gel with the incorporation of adequate amounts of SOFE resulted in an improvement of the mechanical properties (Yilmaz and Ediz 2008; Alcamand et al. 2018). Excessive amount of calcium has a negative effect on the mechanical properties of alkaline-activated cements due to the lower amount of strong Si–O-Al bonds in the geopolymer system (Yaseri et al. 2017).

The hydration time has a great influence when SOFE is incorporated, obtaining lower values of flexural and compression strength after 7 days of curing than the control alkaline-activated cement. This may be due to the fact that SOFE, because of their more porous structure, absorb more water (Lo and Cui 2004) and have a slower activation mechanism which results in a later hardening of the binder requiring longer curing times to achieve higher flexural and compression strengths.

These results are in agreement with those obtained by other authors. Sabbas et al., (2023) studied the mechanical activation of one-part slag-based geopolymers with 10–30 wt% RHA substitution activated with NaOH and sodium metasilicate. The authors obtained strength values to decreased with the incorporation of RHA after 7 days of curing while it increased at 28 days of curing with the incorporation of up to 20 wt% RHA due to the high SiO₂/Al₂O₃ ratio, as well as the high porosity and reactivity of the silica present in RHA that favors the formation of additional calcium silicate hydrate (C-S–H) phases in geopolymeric systems containing calcium oxide, given the pozzolanic activity that RHA exhibits in the presence of Ca^2^⁺ ions (Hossain et al. 2021). Cheng et al. (2024) obtained optimum flexural and compressive strength values of 6.4 MPa and 36.3 MPa, respectively, with the addition of 20 wt% of calcined diatomaceous earth to mortars based on blast furnace slags after 28 days of curing. A 20 wt% is indicated as the optimum substitution rate to balance strength and workability due to increased C-(A)-S–H gel formation due to the high amorphous silica content in the diatomaceous earths that reacts with slag and alkali.

The results of pH evolution over time are shown in Table 5. The incorporation of SOFE affects the alkalinity of the system and its evolution during the curing process. Initially, all alkaline-activated mixtures present pH values above 13.20, with a slight increase as the amount of SOFE increases, reaching maximum values of 13.44 in the sample with 50 wt% SOFE (EAFS- 50SOFE). This is common in the early stages of curing when hydrogen ions (H⁺) are released due to the dissolution of silica, alumina, and calcium from the precursors. However, with the passage of time, a general trend of decreasing pH is observed in all cements, which may be an indication of the formation of C-A-S–H and N-A-S–H, which are more stable and less alkaline structures. This decrease is more pronounced at 7 days in the control sample without SOFE (EAFS- 0SOFE), which reaches a pH of 12.01. In contrast, the samples with SOFE maintain a higher pH during the first few days, although they also eventually tend to decrease after 7 days of curing; however, a slower decrease in pH was observed in the mixtures as the SOFE content increases, which correlates with delayed geopolymerization and a lower initial dissolution of reactive components and a slower reaction which justifies the lower mechanical strength at the 7-day curing age.

**Table 5:** Evolution of pH with time for alkaline activation cements

Evolution of pH with the time							
Sample	1 h	3 h	8 h	1 d	3 d	7 d	28 d
EAFS- 0SOFE	13.20	13.00	13.03	12.97	12.61	12.01	11.90
EAFS- 10SOFE	13.35	13.32	13.25	13.12	13.26	12.26	12.09
EAFS- 20SOFE	13.37	13.18	13.19	13.14	13.24	12.44	12.22
EAFS- 30SOFE	13.36	13.17	13.29	13.16	13.25	12.78	11.79
EAFS- 40SOFE	13.38	13.36	13.30	13.18	13.25	12.72	11.89
EAFS- 50SOFE	13.44	13.27	13.22	13.23	13.32	12.80	11.70

### Thermal conductivity

**Fig. 12:**
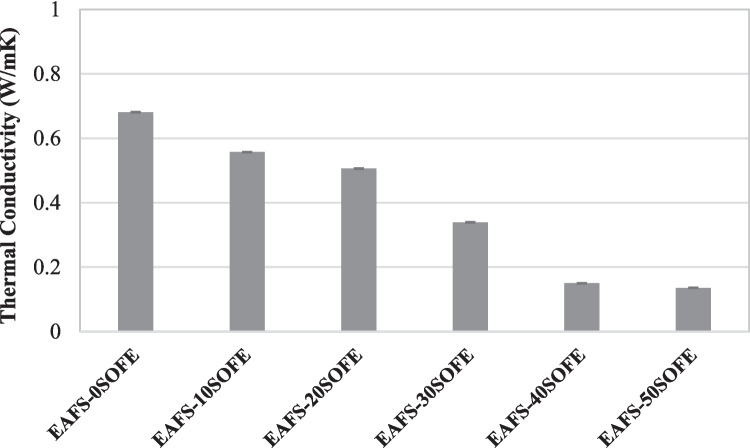
Thermal conductivity of EAFS-xSOFE alkaline-activated cements at 28 days of curing

Thermal conductivity indicates the capability of a material to transfer heat, so a lower thermal conductivity value suggests better insulation (Novais et al. 2016). In general terms, alkaline-activated geopolymers or cements have a thermal conductivity lower than 0.70 W/mK (Subaer and van Riessen 2007), which is approximately 50% lower than that of Portland cement (PC) materials (Fongang et al. 2015). Figure 12 shows the thermal conductivity results for each binder as a function of the percentage substitution of EAFS for SOFE at 28 days of curing. The thermal conductivity of the EAFS- 0SOFE control cement is 0.681 W/mK, with the thermal conductivity decreasing significantly to 0.136 W/mK for the EAFS- 50SOFE binders, which represents a decrease of 80% with respect to the control cement. As the percentage of SOFE substitution increases, more insulating alkaline activation cements are obtained, according to bulk density and total porosity data. The geopolymerization reaction produced interconnected amorphous and porous polysialates that provided a tortuous path for thermal gradient flow (He et al. 2013). The amorphous and porous structure within the geopolymer network acted as an effective barrier restricting heat flow. The higher conductivity of the samples containing higher amounts of EAFS exhibiting higher thermal conductivity values may be due to the higher chemically bound water content providing a more continuous gel structure and the lower porosity (Pan et al. 2013) as deduced by the SEM study.

### Leaching test

The use of industrial by-products requires rigorous evaluation and analysis to ensure environmental risk control. For this reason, alkaline activation cements have been tested for leaching in accordance with EN 12457–1 (EN 12457–1, 2003), and the values obtained have been compared with the leaching limit values for inert, non-hazardous, and hazardous waste admissible in landfills for liquid to solid ratios of 2 L/kg, set by Decision 2003/33/EC of the Council of the European Union (Council of the European Union, 2003) (Table 6). In all the formulations analyzed, the concentrations of heavy metals remained below the limits established for non-hazardous wastes. Most of the elements met even the strictest requirements for inert waste, which is evidence of the chemical stability of the materials developed and their low potential for metal release under standard leaching conditions. Elements such as arsenic (As), barium (Ba), cadmium (Cd), chromium (Cr), copper (Cu), nickel (Ni), lead (Pb), and zinc (Zn) presented values below the limits for inert wastes in all alkaline-activated cements. For example, As concentrations ranged from 0.0096 to 0.1500 mg/L, always remaining below the 0.17 mg/L limit. Similarly, Cd showed very low values (≤ 0.0004 mg/L) versus the 0.003 mg/L threshold for inert waste. In the case of mercury (Hg), concentrations were close to the limit for inert residues (0.003 mg/L) in the control sample without SOFE (EAFS- 0SOFE) (0.0033 mg/L), although all other formulations were below this value. On the other hand, molybdenum (Mo) slightly exceeded the limit for inert waste (0.3 mg/L) in the samples with 40 wt% and 50 wt% SOFE; however, the concentrations remained well below the limits for non-hazardous waste (5 mg/L), guaranteeing the non-hazardousness of the material. As for antimony (Sb) and selenium (Se), the control sample without SOFE (EAFS- 0SOFE) presented a concentration of 0.043 mg/L and 0.1941 mg/L, respectively, which exceeds the threshold for inert waste (0.02 and 0.06 mg/L, respectively) but remains below the limit for non-hazardous waste (0.02 and 0.3 mg/L, respectively). As the proportion of SOFE increased, the concentrations of Sb and Se decreased markedly, reaching values below the limit for inert residues from EAFS- 20SOFE (0.0014 and 0.0595 mg/L). In formulations with 30 wt% or more SOFE, the values were significantly lower, ensuring minimal environmental impact.

**Table 6:** Leachability of heavy metals of alkaline activation cements in mg/L and EU limit values (mg/kg-assay 2 L/kg) for waste admissible in landfills as inert waste, non-hazardous waste, and hazardous waste

mg/L	EAFS- 0SOFE	EAFS- 10SOFE	EAFS- 20SOFE	EAFS- 30SOFE	EAFS- 40SOFE	EAFS- 50SOFE	Inert waste*	Non-hazardous waste*	Hazardous waste*
As	0.1500	0.0096	0.145	0.130	0.107	0.0657	0.17	0.4	0.4
Ba	0.1499	0.1068	0.1855	0.2111	0.3813	0.6879	7	10	30
Cd	0.0004	0.0003	0.0004	0.0004	0.0004	0.0003	0.003	0.6	0.6
Cr	0.1232	0.0346	0.0203	0.0162	0.0156	0.0179	0.2	4	4
Cu	0.0029	0.0013	0.0014	0.0015	0.0023	0.0024	0.9	25	25
Hg	0.0033	0.0030	0.0028	0.0025	0.0018	0.0011	0.003	0.05	0.05
Mo	0.123	0.203	0.235	0.248	0.328	0.338	0.3	5	5
Ni	0.0018	0.0009	0.0008	0.0007	0.0009	0.0011	0.2	5	5
Pb	0.0006	0.0004	0.0007	0.0008	0.0014	0.0019	0.2	5	5
Sb	0.0043	0.0026	0.0014	0.0009	0.0005	0.0004	0.02	0.2	0.2
Se	0.1941	0.1092	0.0595	0.0514	0.0048	0.0041	0.06	0.3	0.3
Zn	0.0061	0.0026	0.0037	0.0040	0.0058	0.0082	2	25	25

*Decision 2003/33/EC of the Council of the European Union

These results demonstrate that the alkaline-activated cements studied do not exceed the regulatory limits for non-hazardous waste, which supports their viability from an environmental perspective. The compliance with the limits for inert waste in most of the analyzed elements reinforces the potential of these materials as a sustainable and safe option for various applications.

## Conclusions

In this work, the technological properties of alkaline-activated cements based on electric arc furnace slags in which 0 − 50 wt% of the EAFS precursor is replaced by the spent oil filtering earths (SOFE) residue are studied.

The following conclusions can be drawn from the test results and analyses providing new insights into the properties of EAFS-SOFE blended cements:

- Increasing the SOFE content leads to a reduction in the bulk density and an increase in water absorption and total porosity of the alkaline-activated cements, which is primarily due to the lower real density of the SOFE residue and the greater liquid/binder ratio required to obtain the adequate initial consistency and workability of every paste.
- The substitution of SOFE for EAFS resulted in an increase in flexural and compressive strength of the binders after 28 days of curing. The inclusion of SOFE, which is rich in silica, promotes the synergetic formation of N-(A)-S–H geopolymeric gel in combination with the C-(A)-S–H gel formed in binders with higher calcium content or with substantial amounts of EAFS, leading to a hybrid N,C-A-S–H gel. The hybrid gel becomes more enriched in sodium as increasing amounts of SOFE are incorporated, as evidenced by SEM–EDS and FTIR studies. It may be responsible for the improved mechanical properties observed in mixed systems that incorporate 30–50 wt% of SOFE.
- The technological properties are notably affected with the hydration time, since the addition of SOFE produced a delay in the geopolymerization reactions increasing notably the bulk density, flexural, and compressive strength and decreasing remarkably the water absorption and total porosity when the curing time is increased from 7 to 28 days.
- The thermal conductivity of EAFS-based alkaline-activated cements decreased with the incorporation of increasing amounts of SOFE up to 0.136 W/mK. Samples incorporating SOFE exhibited a more porous structure that improved the thermal insulation properties.
- The results obtained confirm the environmental viability of the alkaline activation cements developed, since all the formulations analyzed complied with the regulatory limits for non-hazardous waste admissible in landfills established by European legislation. Most of the elements evaluated presented concentrations below the thresholds for inert waste, which evidences the chemical stability of the materials and their low potential for metal release under standard leaching conditions.

Hence, the research underscores the beneficial impact of employing a waste material like spent oil filtering earths (SOFE), which typically has limited industrial applications, as a replacement for the precursor electric arc steel slag (EAFS) in the production of alkaline activation cements. This substitution results in binders with enhanced physical, mechanical, and thermal properties compared to standard control cements.

## Data Availability

Data are available on request from the authors.

## References

[CR1] Alcamand HA, Borges PH, Silva FA, Trindade ACC (2018) The effect of matrix composition and calcium content on the sulfate durability of metakaolin and metakaolin/slag alkali-activated mortars. Ceram Int 44(5):5037–5044. 10.1016/j.ceramint.2017.12.102

[CR2] Amaral MM, Yukuhiro VY, Vicentini R, Peterlevitz AC, Da Silva LM, Fernandez P, Zanin H (2022) Direct observation of the CO2 formation and C-H consumption of carbon electrode in an aqueous neutral electrolyte supercapacitor by in-situ FTIR and Raman. J Energy Chem 71:488–496. 10.1016/j.jechem.2022.03.020

[CR3] ASTM C1437–20 (2020) Standard test method for flow of hydraulic cement mortar. West Conshohocken: ASTM International

[CR4] Bakharev T, Sanjayan JG, Cheng YB (1999) Alkali activation of Australian slag cements. Cem Concr Res 29:113–120. 10.1016/S0008-8846(98)00170-7

[CR5] Balaguera CAC, Botero MAG (2020) Characterization of steel slag for the production of chemically bonded phosphate ceramics (CBPC). Constr Build Mater 241:118138. 10.1016/j.conbuildmat.2020.118138

[CR6] Bernal S, Gutierrez RD, Delvasto S, Rodriguez E (2010) Performance of an alkali activated slag concrete reinforced with steel fibers. Constr Build Mater 24:208–214. 10.1016/j.conbuildmat.2007.10.027

[CR7] Bernal SA, de Gutiérrez RM, Pedraza AL, Provis JL, Rodriguez ED, Delvasto S (2011) Effect of binder content on the performance of alkali-activated slag concretes. Cem Concr Res 41(1):1–8. 10.1016/j.cemconres.2010.08.017

[CR8] Boey PL, Ganesan S, Maniam GP, Ali DMH (2011) Ultrasound aided in situ transesterification of crude palm oil adsorbed on spent bleaching clay. Energy Convers Manage 52(5):2081–2084. 10.1016/j.enconman.2010.12.013

[CR9] Bouaissi A, Li LY, Abdullah MMAB, Bui QB (2019) Mechanical properties and microstructure analysis of FA-GGBS-HMNS based geopolymer concrete. Constr Build Mater 210:198–209. 10.1016/j.conbuildmat.2019.03.202

[CR10] Catauro M, Bollino F, Papale F, Lamanna G (2014) Investigation of the sample preparation and curing treatment effects on mechanical properties and bioactivity of silica rich metakaolin geopolymer. Mater Sci Eng, C 36:20–24. 10.1016/j.msec.2013.11.026 10.1016/j.msec.2013.11.026 24433882

[CR11] Chen Z, Li JS, Zhan BJ, Sharma U, Poon CS (2018) Compressive strength and microstructural properties of dry-mixed geopolymer pastes synthesized from GGBS and sewage sludge ash. Constr Build Mater 182:597–607. 10.1016/j.conbuildmat.2018.06.159

[CR12] Cheng X, Zhang H, Li W, Zhang L (2024) Utilizing diatomaceous earth (DE) as a sustainable substitute in alkali-activated cementitious materials: performance and life cycle assessment. Constr Build Mater 452:138889. 10.1016/j.conbuildmat.2024.138889

[CR13] Chi M, Huang R (2013) Binding mechanism and properties of alkali-activated fly ash/slag mortars. Constr Build Mater 40:291–298. 10.1016/j.conbuildmat.2012.11.003

[CR14] Coelho TPP, Bezerra BP, Verza JR, Luz AP, Morelli MR (2023) Physico-mechanical properties of metakaolin and diatomite-based geopolymers. Mater Lett 349:134784. 10.1016/j.matlet.2023.134784

[CR15] Collins F, Sanjayan JG (1999) Strength and shrinkage properties of alkali-activated slag concrete containing porous coarse aggregate. Cem Concr Res 29:607–610. 10.1016/S0008-8846(98)00203-8

[CR16] Collins F, Sanjayan JG (2000) Cracking tendency of alkali-activated slag concrete subjected to restrained shrinkage. Cem Concr Res 30:791–798. 10.1016/S0008-8846(00)00243-X

[CR17] Council of the European Union (2003) Council Decision C of 19 December 2002 establishing criteria and procedures for the acceptance of waste at landfills pursuant to Article 16 of and Annex II to Directive 1999/31/EC. Off J Eur Communities L 11(2003):27–49

[CR18] Davidovits J (1989) Geopolymers and geopolymeric materials. J Therm Anal 35(2):429–441. 10.1007/BF01904446

[CR19] Dong B, Chen C, Fang G, Wu K, Wang Y (2022) Positive roles of lime mud in blended Portland cement. Constr Build Mater 328:127067. 10.1016/j.conbuildmat.2022.127067

[CR20] EN 1015–10:2020 (2020) Methods of test for mortar for masonry–Part 10: determination of dry bulk density of hardened mortar

[CR21] EN 1015–11:2020 (2000) Methods of test for mortar for masonry–Part 11: determination of flexural and compressive strength of hardened mortar

[CR22] EN 12457–1:2003 (2003) Characterisation of waste - leaching - compliance test for leaching of granular waste materials and sludges - Part 1: one stage batch test at a liquid to solid ratio of 2 l/kg for materials with high solid content and with particle size below 4 mm (without or with size reduction)

[CR23] Felaous K, Aziz A, Achab M (2023) Physico-mechanical and durability properties of new eco-material based on blast furnace slag activated by Moroccan diatomite gel. Environ Sci Pollut Res 30(2):3549–3561. 10.1007/s11356-022-22461-7 10.1007/s11356-022-22461-7 35948795

[CR24] Fine G, Stolper E (1986) Dissolved carbon dioxide in basaltic glasses: concentrations and speciation. Earth Planet Sci Lett 76(3–4):263–278. 10.1016/0012-821X(86)90078-6

[CR25] Fongang RT, Pemndje J, Lemougna PN, Melo UC, Nanseu CP, Nait-Ali B, Kamseu E, Leonelli C (2015) Cleaner production of the lightweight insulating composites: microstructure, pore network and thermal conductivity. Energy Build 107:113–122. 10.1016/j.enbuild.2015.08.009

[CR26] García-Lodeiro I, Palomo A, Fernández-Jiménez A, Macphee DE (2011) Compatibility studies between NASH and CASH gels. Study in the ternary diagram Na_2_O–CaO–Al_2_O_3_–SiO_2_–H_2_O. Cem Concr Res 41(9):923–931. 10.1016/j.cemconres.2011.05.006

[CR27] Gómez-Casero MA, Pérez-Villarejo L, Sánchez-Soto PJ, Eliche-Quesada D (2022) Comparative study of alkali activated cements based on metallurgical slags, in terms of technological properties developed. Sustainable Chem Pharm 29:100746. 10.1016/j.scp.2022.100746

[CR28] Hassan HS, Abdel-Gawwad HA, Vásquez-García SR, Israde-Alcántara I, Flores-Ramirez N, Rico JL, Mohammed MS (2019) Cleaner production of one-part white geopolymer cement using pre-treated wood biomass ash and diatomite. J Cleaner Prod 209:1420–1428. 10.1016/j.jclepro.2018.11.137

[CR29] He P, Jia D, Wang S (2013) Microstructure and integrity of leucite ceramic derived from potassium-based geopolymer precursor. J Eur Ceram Soc 33(4):689–698. 10.1016/j.jeurceramsoc.2012.10.019

[CR30] Hossain SS, Roy PK, Bae C-J (2021) Utilization of waste rice husk ash for sustainable geopolymer: a review. Const Build Mater 310:125218. 10.1016/j.conbuildmat.2021.125218

[CR31] Hu X, Shi C, Shi Z, Zhang L (2019) Compressive strength, pore structure and chloride transport properties of alkali-activated slag/fly ash mortars. Cem Concr Compos 104:103392. 10.1016/j.cemconcomp.2019.103392

[CR32] Hui-Teng N, Cheng-Yong H, Yun-Ming L, Abdullah MMAB, Hun KE, Razi HM, Yong-Sing N (2021) Formulation, mechanical properties and phase analysis of fly ash geopolymer with ladle furnace slag replacement. J Mater Res Technol 12:1212–1226. 10.1016/j.jmrt.2021.03.065

[CR33] ISO 8302:1991 (1991) Thermal insulation - determination of steady-state thermal resistance and related properties - guarded hot plate apparatus

[CR34] Jena S, Panigrahi R (2021) Feasibility study of the properties of geopolymer concrete with ferrochrome slag and silica fume. Materials Today: Proceedings 38:2476–2480. 10.1016/j.matpr.2020.07.510

[CR35] Khale D, Chaudhar R (2007) Mechanism of geopolymerization and factors influencing its development: a review. J Mater Sci 42:729–746. 10.1007/s10853-006-0401-4

[CR36] Khan MSH, Castel A, Akbarnezhad A, Foster SJ, Smith M (2016) Utilisation of steel furnace slag coarse aggregate in a low calcium fly ash geopolymer concrete. Cem Concr Res 89:220–229. 10.1016/j.cemconres.2016.09.001

[CR37] Kim HS, Kim KS, Jung SS, Hwang JI, Choi JS, Sohn I (2015) Valorization of electric arc furnace primary steelmaking slags for cement applications. Waste Manage 41:85–93. 10.1016/j.wasman.2015.03.019 10.1016/j.wasman.2015.03.019 25863765

[CR38] Liang G, Yao W (2023) Effect of diatomite on the reaction kinetics, early-age chemical shrinkage and microstructure of alkali-activated slag cements. Constr Build Mater 376:131026. 10.1016/j.conbuildmat.2023.131026

[CR39] Liu Y, Yang Z, Qu S, Li Y, Ju X, Cui B, Du Z, Ali A, Wang D, Chen Z, Zhou A (2025) Study on microcrystalline structure and model construction of semi-coke based on XRD, XPS, FTIR, 13C NMR, and HRTEM. J Mol Struct 141264. 10.1016/j.molstruc.2024.141264

[CR40] Lo TY, Cui HZ (2004) Effect of porous lightweight aggregate on strength of concrete. Mater Lett 58(6):916–919. 10.1016/j.matlet.2003.07.036

[CR41] Mehta A, Siddique R (2018) Sustainable geopolymer concrete using ground granulated blast furnace slag and rice husk ash: strength and permeability properties. J Clean Prod 205:49–57. 10.1016/j.jclepro.2018.08.313

[CR42] Mendioroz S, Belzunce MJ, Pajares JA (1989) Thermogravimetric study of diatomites. J Therm Anal 35:2097–2104. 10.1007/bf01911874

[CR43] Mir N, Khan SA, Kul A, Sahin O, Lachemi M, Sahmaran M, Koç M (2022) Life cycle assessment of binary recycled ceramic tile and recycled brick waste-based geopolymers. Clean Mater 5:100116. 10.1016/j.clema.2022.100116

[CR44] Mishra A, Lahoti M (2023) Effect of sodium hydroxide concentration on EAFS based alkali activated binder. Mater Today Proc. 10.1016/j.matpr.2023.03.717

[CR45] Mishra A, Lahoti M, Yang EH (2023) Mitigating environmental impact by development of ambient-cured EAF slag and fly ash blended geopolymer via mix design optimization. Environ Sci Pollut Res 1–18. 10.1007/s11356-023-26884-8 10.1007/s11356-023-26884-8 37103709

[CR46] MITECO (Ministerio para la transformación ecológica y el reto demográfico) (2021) Análisis de la aplicación del concepto de fin de condición de residuo de las escorias de fundición de horno de arco eléctrico-acero al carbono, cobre y silicomanganeso- para su uso como árido (en aplicaciones ligadas y no ligadas) y otros usos: como materia prima en la fabricación de productos de construcción (cemento y clínker) y como material abrasivo [PDF file].https://www.miteco.gob.es/content/dam/miteco/images/es/estudio_fcr_escorias_vdef_julio21_tcm30-529624.pdf. Accessed Jul 2024

[CR47] Morrow BA, McFarlan AJ (1992) Surface vibrational modes of silanol groups on silica. J Phys Chem 96(3):1395–1400. 10.1021/j100182a068

[CR48] Nguyen H, Nguyen TD, Tran TVN, Nguyen DL, Tran HS, Nguyen TL, Nguyen TH, Nguyen HG (2022) Steel slag quality control for road construction aggregates and its environmental impact: case study of Vietnamese steel industry—leaching of heavy metals from steel-making slag. Environ Sci Pollut Res 29:41983–41991. 10.1007/s11356-021-16438-1 10.1007/s11356-021-16438-1 34564812

[CR49] Nikolić I, Đurović D, Marković S, Veselinović L, Janković-Častvan I, Radmilović VV, Radmilović VR (2020) Alkali activated slag cement doped with Zn-rich electric arc furnace dust. J Mater Res Technol 9(6):12783–12794. 10.1016/j.jmrt.2020.09.024

[CR50] Nikolić I, Marković S, Janković-Častvan I, Radmilović VV, Karanović L, Babić B, Radmilović VR (2016) Modification of mechanical and thermal properties of fly ash-based geopolymer by the incorporation of steel slag. Mater Lett 176:301–305. 10.1016/j.matlet.2016.04.121

[CR51] Nikolov A, Nugteren H, Rostovsky I (2020) Optimization of geopolymers based on natural zeolite clinoptilolite by calcination and use of aluminate activators. Constr Build Mater 243:118257. 10.1016/j.conbuildmat.2020.118257

[CR52] Novais RM, Buruberri LH, Ascensão G, Seabra MP, Labrincha JA (2016) Porous biomass fly ash-based geopolymers with tailored thermal conductivity. J Cleaner Prod 119:99–107. 10.1016/j.jclepro.2016.01.083

[CR53] Oyebisi S, Ede A, Olutoge F, Ogbiye S (2020) Evaluation of reactivity indexes and durability properties of slag-based geopolymer concrete incorporating corn cob ash. Constr Build Mater 258:119604. 10.1016/j.conbuildmat.2020.119604

[CR54] Ozturk M, Bankir MB, Bolukbasi OS, Sevim UK (2019) Alkali activation of electric arc furnace slag: mechanical properties and micro analyzes. J Build Eng 21:97–105. 10.1016/j.jobe.2018.10.005

[CR55] Palomo A, Grutzeck MW, Blanco MT (1999) Alkali-activated fly ashes: a cement for the future. Cem Concr Res 29(8):1323–1329. 10.1016/S0008-8846(98)00243-9

[CR56] Pan Z, Feng KN, Gong K, Zou B, Korayem AH, Sanjayan J, Duan WH, Collins F (2013) Damping and microstructure of fly ash-based geopolymers. J Mater Sci 48:3128–3137. 10.1007/s10853-012-7090-y

[CR57] Part WK, Ramli M, Cheahw CB (2015) An overview on the influence of various factors on the properties of geopolymer concrete derived from industrial byproducts. Constr Build Mater 77:370–395. 10.1016/j.conbuildmat.2014.12.065

[CR58] Phoo-Ngernkham T, Hanjitsuwan S, Damrongwiriyanupap N, Chindaprasirt P (2017) Effect of sodium hydroxide and sodium silicate solutions on strengths of alkali activated high calcium fly ash containing Portland cement. KSCE J Civ Eng 21:2202–2210. 10.1007/s12205-016-0327-6

[CR59] Puertas F, Martínez-Ramírez S, Alonso S, Vázquez T (2000) Alkali-activated fly ash/slag cements: strength behaviour and hydration products. Cem Concr Res 30(10):1625–1632. 10.1016/S0008-8846(00)00298-2

[CR60] Puligilla S, Mondal P (2015) Co-existence of aluminosilicate and calcium silicate gel characterized through selective dissolution and FTIR spectral subtraction. Cem Concr Res 70:39–49. 10.1016/J.CEMCONRES.2015.01.006

[CR61] Rashad AM, Khafaga SA, Gharieb M (2021) Valorization of fly ash as an additive for electric arc furnace slag geopolymer cement. Constr Build Mater 294:123570. 10.1016/j.conbuildmat.2021.123570

[CR62] Rojas N, Bustamante M, Muñoz P, Godoy K, Letelier V (2023) Study of properties and behavior of concrete containing EAF slag as coarse aggregate. Dev Built Environ 14:100137. 10.1016/j.dibe.2023.100137

[CR63] Sabbar Abbas I, Hamid Abed M, Canakci H (2023) Development and characterization of eco- and user-friendly grout production via mechanochemical activation of slag/rice husk ash geopolymers. J Build Eng 63 (Part A):105336. 10.1016/j.jobe.2022.105336

[CR64] Salas DA, Ramirez AD, Ulloa N, Baykara H, Boero AJ (2018) Life cycle assessment of geopolymer concrete. Constr Build Mater 190:170–177. 10.1016/j.conbuildmat.2018.09.123

[CR65] Somna K, Jaturapitakkul C, Kajitvichyanukul P, Chindaprasirt P (2011) NaOH-activated ground fly ash geopolymer cured at ambient temperature. Fuel 90(6):2118–2124. 10.1016/j.fuel.2011.01.018

[CR66] Subaer n, van Riessen A (2007) Thermo-mechanical and microstructural characterisation of sodium-poly (sialate-siloxo) (Na-PSS) geopolymers. J Mater Sci 42:3117–3123. 10.1007/s10853-006-0522-9

[CR67] Sukmak P, Sukmak G, De Silva P, Horpibulsuk S, Kassawat S, Suddeepong A (2023) The potential of industrial waste: electric arc furnace slag (EAF) as recycled road construction materials. Constr Build Mater 368:130393. 10.1016/j.conbuildmat.2023.130393

[CR68] Tho-In T, Sata V, Boonserm K, Chindaprasirt P (2018) Compressive strength and microstructure analysis of geopolymer paste using waste glass powder and fly ash. J Cleaner Prod 172:2892–2898. 10.1016/j.jclepro.2017.11.125

[CR69] Walkley B, San NR, Sani MA, Rees GJ, Hanna JV, van Deventer JS, Provis JL (2016) Phase evolution of C-(N)-ASH/NASH gel blends investigated via alkali-activation of synthetic calcium aluminosilicate precursors. Cem Concr Res 89:120–135. 10.1016/j.cemconres.2016.08.010

[CR70] Worldsteel Association (2021) Steel Industry CoProducts. https://worldsteel.org/wp-content/uploads/Fact-sheet-Steel-industry-co-products.pdf. Accessed May and Sept 2024

[CR71] Yaseri S, Hajiaghaei G, Mohammadi F, Mahdikhani M, Farokhzad R (2017) The role of synthesis parameters on the workability, setting and strength properties of binary binder based geopolymer paste. Constr Build Mater 157:534–545. 10.1016/j.conbuildmat.2017.09.102

[CR72] Yılmaz B, Ediz N (2008) The use of raw and calcined diatomite in cement production. Cem Concr Compos 30(3):202–211. 10.1016/j.cemconcomp.2007.08.003

[CR73] Yip CK, Van Deventer JSJ (2003) Microanalysis of calcium silicate hydrate gel formed within a geopolymeric binder. J Mater Sci 38:3851–3860. 10.1023/A:1025904905176

[CR74] Zhang H (2011) Cement. In: Building materials in civil engineering. Woodhead Publishing, pp 46–80. 10.1533/9781845699567.46

[CR75] Zhao YH, Geng JT, Cai JC, Cai YF, Cao CY (2020) Adsorption performance of basic fuchsin on alkali-activated diatomite. Adsorpt Sci Technol 38(5–6):151–167. 10.1177/0263617420922084

