# Correction to: Impact of incorporating spent oil filtering earths into the formulation of alkali‑activated cements based on electric arc furnace slag

**DOI:** 10.1007/s11356-025-36504-2

**Published:** 2025-05-09

**Authors:** Pedro Delgado‑Plana, Miguel Ángel Gómez‑Casero, Salvador Bueno‑Rodríguez, Pedro José Sánchez‑Soto, Dolores Eliche‑Quesada

**Affiliations:** 1 Department of Chemical, Environmental, and Materials Engineering, Higher Polytechnic School of Jaén, University of Jaen, Campus Las Lagunillas S/N, 23071 Jaén, Spain; 2 Center for Advanced Studies in Earth Sciences, Energy and Environment (CEACTEMA), University of Jaén, Campus Las Lagunillas, S/N, 23071 Jaén, Spain; 3 Institute of Materials Science of Sevilla, Joint Center of CSIC (Spanish National Research Council) and University of Sevilla, 41092 Seville, Spain

**Correction to: Environmental Science and Pollution Research**

10.1007/s11356-025-36429-w

The caption of Figure 4 is incorrect.

It should be “Figure 4. FTIR analysis of EAFS and SOFE as raw material and after thermal treatment”

The text "The morphology of the EAFS and SOFE particles was observed by scanning electron microscope (SEM) (Figure 5) using a Carl Zeiss model Merlin assisted by Energy Dispersive X-ray Spectroscopy (EDS). The samples were mounted on an aluminium grid and subsequently coated with carbon using the JEOL JFC 1100 sputter coater. It can be seen that both precursors present particles in a wide range of sizes. The EAFS presents an irregular shape, with a predominance of angular particles. EDS analysis showed the presence of calcium, iron, silica, aluminum magnesium and manganese (Spectrum 1). In the SOFE residue different shapes can be identified with predominance of a rough surface texture with porous microstructure which is the reason why it is used as a filter. In this case, EDS analysis indicated a predominance of silica with a lower content of aluminum and other components such as sodium, potassium, iron and calcium (Spectrum 2). These EDS results agree with data presented in Table 1." should be placed before the heading "Test plan"

The Original article has been corrected.

